# Invasive Candidiasis in Various Patient Populations: Incorporating Non-Culture Diagnostic Tests into Rational Management Strategies

**DOI:** 10.3390/jof2010010

**Published:** 2016-02-06

**Authors:** Cornelius J. Clancy, Ryan K. Shields, M. Hong Nguyen

**Affiliations:** 1 VA Pittsburgh Healthcare System, Division of Infectious Diseases, University of Pittsburgh, Scaife Hall 867, 3550 Terrace St., Pittsburgh, PA 15261, USA; 2 Department of Medicine, Division of Infectious Diseases, University of Pittsburgh, Scaife Hall 871, 3550 Terrace St., Pittsburgh, PA 15261, USA; shieldsrk@upmc.edu

**Keywords:** invasive candidiasis, candidemia, intra-abdominal candidiasis, β-d-glucan, polymerase chain reaction (PCR), diagnosis, Bayesian

## Abstract

Mortality rates due to invasive candidiasis remain unacceptably high, in part because the poor sensitivity and slow turn-around time of cultures delay the initiation of antifungal treatment. β-d-glucan (Fungitell) and polymerase chain reaction (PCR)-based (T2Candida) assays are FDA-approved adjuncts to cultures for diagnosing invasive candidiasis, but their clinical roles are unclear. We propose a Bayesian framework for interpreting non-culture test results and developing rational patient management strategies, which considers test performance and types of invasive candidiasis that are most common in various patient populations. β-d-glucan sensitivity/specificity for candidemia and intra-abdominal candidiasis is ~80%/80% and ~60%/75%, respectively. In settings with 1%–10% likelihood of candidemia, anticipated β-d-glucan positive and negative predictive values are ~4%–31% and ≥97%, respectively. Corresponding values in settings with 3%–30% likelihood of intra-abdominal candidiasis are ~7%–51% and ~78%–98%. β-d-glucan is predicted to be useful in guiding antifungal treatment for wide ranges of populations at-risk for candidemia (incidence ~5%–40%) or intra-abdominal candidiasis (~7%–20%). Validated PCR-based assays should broaden windows to include populations at lower-risk for candidemia (incidence ≥~2%) and higher-risk for intra-abdominal candidiasis (up to ~40%). In the management of individual patients, non-culture tests may also have value outside of these windows. The proposals we put forth are not definitive treatment guidelines, but rather represent starting points for clinical trial design and debate by the infectious diseases community. The principles presented here will be applicable to other assays as they enter the clinic, and to existing assays as more data become available from different populations.

## 1. Introduction

Candidemia and other types of invasive candidiasis carry mortality rates of 25%–40% [1]. Prompt antifungal treatment improves outcomes [2,3], but gold standard diagnostic tests (blood or deep-tissue site cultures) are ~50% sensitive, have turn-around times of several days, and often turn positive late in the disease [4]. Deep-tissue cultures are further limited by need for invasive procedures, which are often contra-indicated due to underlying medical conditions. Poor outcomes and shortcomings of cultures promote antifungal therapy in the absence of conclusive diagnoses, a practice of unclear benefit in many settings that may impact institutional ecology, antifungal resistance and pharmacy budgets [4,5]. Non-culture diagnostics such as β-d-glucan or polymerase chain reaction (PCR) assays detect Candida cellular components in blood or serum, rather than viable organisms. Used judiciously as adjuncts to cultures, these tests can identify more patients with invasive candidiasis, at earlier disease stages [4]. However, there is uncertainty about the performance of non-culture diagnostics in routine practice, and their roles in patient management.

In this paper, we present a paradigm for incorporating non-culture tests into antifungal treatment strategies. In developing these strategies, we review the types of invasive candidiasis, performance of β-d-glucan and PCR assays, and interpretation of test results among different patient groups. We describe a Bayesian framework for applying test results to patient management, and suggest settings in which non-culture diagnostics may be most useful. The proposals we put forth are not definitive treatment guidelines, but rather starting points for discussions and debate. The principles presented here will be applicable to other assays as they enter the clinic, and to existing assays as more data become available from different populations.

## 2. Invasive Candidiasis in Various Populations

Invasive Candida infections comprise candidemia and deep-seated candidiasis (submucosal tissue and organ infections), diseases that may occur concurrently or independently. Approximately 50% of primary candidemia results in secondary deep-seated candidiasis due to hematogenous seeding [4]. Primary deep-seated candidiasis stems from non-hematogenous introduction of Candida into sterile sites, most commonly the abdominal cavity. Studies suggest that <20% of primary deep-seated candidiasis is associated with secondary candidemia [6,7,8]. As detailed below, diagnostic test performance may differ for candidemia and deep-seated candidiasis. Therefore, it is impossible to critically assess studies of non-culture tests or interpret results at the bedside without considering the type of invasive candidiasis that is being diagnosed. We will focus on candidemia and intra-abdominal candidiasis.

Candidemia and intra-abdominal candidiasis are believed to occur with equal frequency [4,9]. The predominant disease is dictated by the clinical setting. In general, candidemia is a low-incidence disease among relatively large at-risk populations. Risk factors are non-specific and common in hospitalized patients, including broad-spectrum antibiotics, intravenous access devices, total parenteral nutrition, mechanical ventilation, renal insufficiency and replacement therapy, diabetes mellitus, corticosteroids, neutropenia or neutrophil dysfunction, and Candida colonization [10]. The incidence of candidemia increases from <1% to ~10% as one moves from any hospitalized patient in whom blood cultures are collected, to low-risk intensive care unit (ICU) patients or patients undergoing non-gastrointestinal (GI) surgery [11,12], to more moderate-risk patients who are ICU residents for ≥4 days or who are in septic shock [13,14], to higher-risk ICU patients identified by clinical prediction rules [13,15,16] (Table 1). In contrast, intra-abdominal candidiasis is a relatively high-incidence disease among more narrowly-defined populations. While patients often have some of the risks above, the disease does not occur in the absence of predisposing factors involving the gastrointestinal (GI) tract or digestive system. Common examples include severe pancreatitis or disruption of GI tract or peritoneal cavity integrity by disease or medical intervention. The incidence of intra-abdominal candidiasis increases from ~3% to ≥30% as one moves from low-to-moderate risk peritoneal dialysis patients with peritonitis [17,18], to high-risk patients with severe acute or necrotizing pancreatitis, or recurrent leaks of the GI tract [8,10,19] (Table 1). In most patients, the predominant type of invasive candidiasis should be apparent when clinicians order a diagnostic test.

**Table 1:** Incidence of invasive candidiasis in various populations.

Risk of IC *	Patient Characteristics	Type of IC **	Incidence ***	References
Low	Any hospitalized patient in whom a blood culture is collected Residence in the ICU without further risk stratification Residence in the ICU post-cardiothoracic surgery	Candidemia	<1%	[11,12,20]
Low-to-moderate	Peritoneal dialysis with peritonitis	Intra-abdominal candidiasis	~3%–6%	[21]
	Presence of septic shock ICU residence for ≥4 days	Candidemia	~3%–7%	[13,14,16]
Moderate	ICU residence for ≥4 days with additional risk factors for IC	Candidemia	~10%–15%	[13,16]
High	Severe acute or necrotizing pancreatitis Recurrent GI track leak requiring surgery	Intra-abdominal candidiasis	~20%–40%	[7,8,19]

IC: Invasive candidiasis; ICU: Intensive care unit; GI: Gastrointestinal; * For descriptive purposes, the follow definitions of level of risk were used: Low: <3%, Low-to-moderate: 3%–10%, Moderate: 10%–20%, High: >20%; ** The most common types of IC are provided. Among patients with candidemia, ~50% will develop secondary deep-seated candidiasis. Among patients with intra-abdominal candidiasis, up to 20% may have secondary candidemia; *** Data are selected from representative publications. Incidence in comparable patient populations may differ by center. In order to best interpret and utilize non-culture test results, clinicians should be aware of the approximate incidence of invasive candidiasis in various settings at their centers.

## 3. Non-Culture Diagnostic Test Performance

Two assays are approved by the U.S. Food and Drug Administration (FDA) as adjuncts to cultures for the diagnosis of invasive candidiasis. The more widely studied test is a serum β-d-glucan assay (Fungitell; Associates of Cape Cod, East Falmouth, MA, USA), which is cleared for diagnosing a variety of invasive fungal infections. The assay does not provide Candida speciation nor distinguish between Candida, Aspergillus and certain other fungi. In meta-analyses of β-d-glucan studies, pooled sensitivity and specificity for invasive candidiasis were 75%–80% and 80%, respectively [21,22]. The more recently approved T2Candida assay (T2 Biosystems, Lexington, MA, USA) uses a self-contained instrument to amplify and detect Candida DNA within whole blood by PCR and T2 magnetic resonance, respectively. FDA approval was based on data from 1501 control patients with Candida-negative blood cultures, and 250 contrived blood specimens spiked with *C. albicans*, *C. glabrata*, *C. parapsilosis*, *C. tropicalis* or *C. krusei* at concentrations ranging from 1 to 100 CFU/mL [20]. The overall sensitivity and specificity were 91% and 99%, respectively. The performance of the assay (in particular, its sensitivity) awaits corroboration, as there are limited published data on whole blood specimens from patients with invasive candidiasis [20,23]. Nevertheless, the preliminary findings are in keeping with meta-analysis of Candida PCR studies, in which pooled sensitivity and specificity for invasive candidiasis were 95% and 92%, respectively [24]. PCR offers potential advantages over β-d-glucan by performing Candida speciation. β-d-glucan may offer advantages among patients in whom aspergillosis and other invasive fungal infections are also considerations.

Definitive interpretation of β-d-glucan and PCR studies is complicated by numerous factors, including heterogeneity in patient and control populations, types of Candida species (or other fungi), testing schedules, definitions of positive test results, and other aspects of research design and data analysis. Meta-analyses have included studies with cohort and case-control designs, in which cases were defined as proven or probable infections and healthy controls were excluded [21,22]. Such studies may overstate performance by excluding possible disease or difficult-to-interpret cases that are commonly encountered in practice. Most studies have enrolled patients with hematologic malignancies or ICU residents. Data are more limited for several important populations at-risk for invasive candidiasis, including solid organ transplant recipients [25]. Studies suggest that β-d-glucan testing of lung and liver transplant recipients may be particularly susceptible to false-positive β-d-glucan results [26,27]. To date, most studies have included a preponderance of patients with candidemia rather than non-bloodstream, deep-seated candidiasis. Therefore, sensitivities and specificities cited above are likely best case scenarios for the diagnosis of candidemia.

The major advantage of non-culture tests over cultures for diagnosing active candidemia may not be improved sensitivity, but rather shorter turn-around times that facilitate earlier treatment. In fact, broth-based blood cultures are quite sensitive at recovering viable Candida from the bloodstream, with median limits of detection of ~1 CFU/mL [28,29]. Isolator blood culture systems may be more sensitive [30,31]. As such, cultures should be positive during the majority of ongoing Candida bloodstream infections. If cultures are negative due to extremely low-level candidemia, the extent to which currently available non-culture tests will make the diagnosis is unclear. Indeed, diagnostic studies of blood culture-positive invasive candidiasis commonly face a “candidemia paradox”, in which non-culture tests appear inferior to a gold standard that is accepted to be suboptimal. For diseases other than candidemia, the biggest clinical impact of a non-culture diagnostic will come from identifying blood culture-negative, primary or secondary deep-seated candidiasis.

In two recent studies, Fungitell β-d-glucan was 53%–65% sensitive and 73%–78% specific for diagnosing deep-seated Candida infections, in particular intra-abdominal candidiasis [6,7]. Blood cultures were only 7%–21% sensitive. Sensitivity and specificity of Candida PCR for deep-seated candidiasis were 89% and 70%, respectively, in one of the studies [6]. β-d-glucan and PCR identified at least some cases of deep-seated candidiasis that were missed by blood cultures, likely by detecting targets released from infected tissues or that persisted within the bloodstream after elimination of viable cells. In both studies, negative controls were at high-risk for intra-abdominal candidiasis. As such, it is very difficult to interpret specificity of the tests, as positive results despite negative cultures may have been false-positives, or true-positives that were missed by cultures. Indeed, uncertainty over how to interpret non-culture test results at the bedside has limited their widespread use.

## 4. Interpreting Non-Culture Tests in Various Populations

No matter how sensitive or specific non-culture assays for invasive candidiasis may be, clinicians must accept a level of uncertainty when interpreting results. By definition, a positive blood culture or tissue culture obtained in a sterile manner establishes the diagnosis of invasive candidiasis. In contrast, non-culture tests are Bayesian biomarkers that assign a probability of disease, which is shaped by the pre-test likelihood, and sensitivity and specificity. Positive and negative predictive values (PPVs, NPVs) can be calculated for β-d-glucan and a PCR assay such as T2Candida in various clinical settings. Examples of anticipated post-test probabilities of invasive candidiasis with positive and negative results for different assays are provided in Table 2.

**Table 2:** Performance of non-culture tests for invasive candidiasis in various populations.

Type of IC	Risk Category	Pre-Test Probability of IC *	Post-Test Probability of IC					
			β-d-Glucan **		PCR ***		Ideal Assay ****	
			Pos.	Neg.	Pos.	Neg.	Pos.	Neg.
Candidemia	Low	1%	4%	<1%	8%	<1%	48%	<1%
	Low-to-moderate	5%	17%	1%	32%	<1%	83%	<1%
	Moderate	10%	31%	3%	50%	1%	91%	1%
Intra-abdominal candidiasis	Low-to-moderate	5%	11%	3%	12%	1%	83%	<1%
	Moderate	10%	21%	6%	23%	3%	91%	1%
	High	30%	51%	22%	53%	11%	97%	4%

PCR: Polymerase chain reaction; Pos: Positive; IC: Invasive candidiasis; Neg: Negative * Pre-test probability of invasive candidiasis is approximated from Table 1; ** Sensitivity/specificity for candidemia and intra-abdominal candidiasis were assumed to be 80%/80% and 60%/75%, respectively; *** Sensitivity/specificity for candidemia and intra-abdominal candidiasis were assumed to be 90%/90% and 80%/70%, respectively; **** Sensitivity/specificity for candidemia and intra-abdominal candidiasis were assumed to be 90%/99% and 90%/99%, respectively.

At low pre-test likelihoods of either disease, PPVs and NPVs are extremely low and extremely high, respectively. As likelihoods increase, PPVs improve at the expense of NPVs. For candidemia, β-d-glucan NPVs remain exceptional (≥97%) in each of the clinical settings; PPV is 31% in the high-risk ICU setting. The superior sensitivity and specificity of PCR are expected to improve PPVs for candidemia compared to β-d-glucan, but the impact on NPVs is likely negligible. If T2Candida is shown to perform as reported in the initial clinical study, PPVs improve further. PPVs and NPVs are lower for intra-abdominal candidiasis than candidemia at a given pre-test likelihood, due to lower sensitivities and specificities. β-d-glucan NPV for intra-abdominal candidiasis is strong (≥97%) in low-risk settings, but values drop to ~80% in higher-risk settings (e.g., severe acute or necrotizing pancreatitis, high-risk GI surgery). β-d-glucan PPV rises to 51% among the highest-risk patients. The superior performance of PCR impacts NPVs in the higher-risk settings, but has less impact on PPVs. There are no data for T2Candida in this setting, but the assay should perform at least as well as PCR.

Table 2 illustrates the Bayesian nature of non-culture diagnostics. The practical challenge is applying these data to the care of patients. How are non-culture diagnostics most likely to be useful in guiding management strategies?

## 5. Integrating Non-Culture Tests into Patient Management Strategies

### 5.1. Screening for Antifungal Treatment

**Figure 1:**
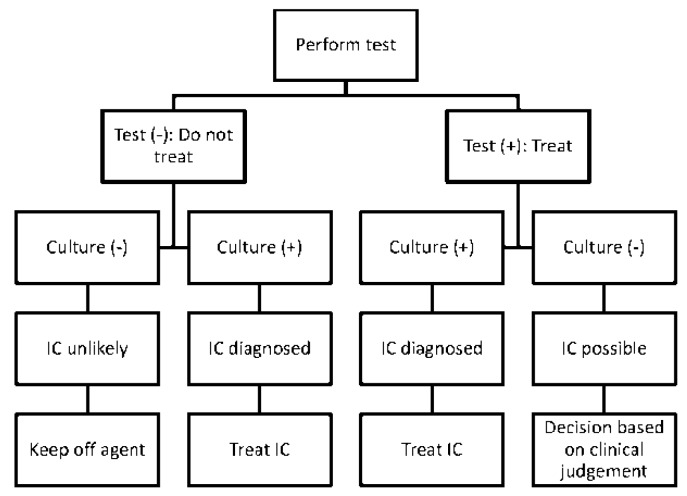
Paradigm for incorporating non-culture tests into antifungal treatment strategies against invasive candidiasis (IC). The paradigm can be applied to pre-emptive or prophylactic antifungal strategies. Treatment decisions are made at two stages, in response to non-culture test results and non-culture results combined with blood cultures, respectively. The viability of the paradigm depends upon positive and negative predictive values (PPVs and NPVs) at each stage. By applying data from Table 2, clinical settings in which non-culture test-driven strategies are likely to be useful can be identified (Table 3).

Since non-culture tests are Bayesian rather than categorical, the interpretation and utilization of results are left to clinical judgment. Broadly speaking, non-culture diagnostics may be used to screen populations for invasive candidiasis, or manage individual patients. At the population level, screening may be incorporated into pre-emptive or prophylactic antifungal strategies, in which treatment is initiated in response to a positive test result or discontinued in response to negative result, respectively. These strategies are attractive conceptually, but they have not been validated conclusively [15,32,33]. A key issue to be resolved for prophylaxis strategies will be the impact of antifungal treatment on test performance. ICUs are attractive settings for interventions based on non-culture screening because empiric and prophylactic antifungal use is wide-spread, populations at-risk for either candidemia or intra-abdominal candidiasis can be identified, and periods of greatest risk can be defined. A paradigm that uses non-culture diagnostics and blood cultures to guide pre-emptive or prophylactic treatment is presented in Figure 1. The paradigm assumes a two-stage decision-making process in which non-culture results are available before concurrently-collected blood cultures. Its feasibility in a given setting will depend upon PPVs and NPVs at each stage.

**Table 3:** Windows in which non-culture tests are predicted to be useful in guiding antifungal treatment.

Predominant Type of IC	Test	Windows ^†^		Comments
		Pre-test Likelihoods	Corresponding Populations	
Candidemia (±DSC)	β-DG ^1^	~5% to 40% *	Low-to-moderate- to high-risk ICU patients	NPVs are ≥~85% at pre-test likelihoods as high as the upper limits of these ranges, suggesting that an antifungal strategy would remain viable. Compared to β-d-glucan, a hypothetical PCR or other ideal assay would broaden the window for antifungal treatment to include lower-risk patients, including those in septic shock. Based on the particulars of a case, clinicians may decide to stop antifungal treatment if blood cultures are negative despite positive non-culture test results. With negative blood cultures, β-d-glucan or PCR PPVs are <15% in all settings with pre-test likelihood <~15%.
	PCR ^2^	~2% to 60% *	Low- to high-risk ICU patients, and patients in septic shock	
	Ideal assay ^3^	~1% to 60% *		
IAC	β-DG ^4^	~7% to 20%	Patients with severe pancreatitiis	Compared to β-d-glucan, a hypothetical PCR assay would broaden the window for antifungal treatment to include high-risk surgery patients with GI leaks, or high-risk liver transplant recipients with bile leaks. An ideal assay for intra-abdominal candidiasis would further broaden the window to include lower-risk patients. Using negative blood culture results to justify withholding pre-emptive treatment or discontinuing prophylaxis among patients with negative β-d-glucan results may broaden the window of pre-test likelihoods to ~7%–30%. Otherwise, negative blood cultures do not significantly impact antifungal strategies.
	PCR ^5^	~7% to 40%	Moderate- to high-risk GI surgery patients, and patients with severe pancreatitis	
	Ideal assay ^3^	~1% to 60%	Peritoneal dialysis patients with peritonitis, in addition to groups above	

IC: Invasive candidiasis; DSC: Deep-seated candidiasis; IAC: Intra-abdominal candidiasis; β-DG: β-d-glucan; PCR: Polymerase chain reaction; ICU: Intensive care unit; NPV: Negative predictive value; PPV: Positive predictive value; GI: Gastrointestinal; ^†^ Windows defined by PPV ≥15% and NPV ≥85%; * In general, clinical prediction models have identified patients with incidence of candidemia up to ~16% [13,16]; ^1^ Sensitivity/specificity: 80%/80% ^2^ 90%/90% ^3^ 90%/99% ^4^ 60%/75% ^5^ 80%/70%.

PPV and NPV thresholds that justify antifungal treatment are not established. Several studies suggest that prophylaxis is beneficial in preventing invasive fungal infections in various settings with baseline rates of disease ≥~15% [34,35,36,37,38,39,40]. As such, it is reasonable to hypothesize that this target is the threshold PPV for initiating pre-emptive antifungal treatment, and a threshold NPV to withhold antifungal treatment is ≥~85%. NPVs beneath this threshold may leave too high a post-test probability of invasive candidiasis for antifungals to be deferred. In order for non-culture screening to be viable, a positive or negative result must provide marginal value beyond simply knowing the pre-test likelihood. In other words, do results sufficiently change the probability of invasive candidiasis such that threshold PPVs and NPVs are achieved? In certain low- and high-risk settings, the answer to this question is no, and screening with non-culture tests will not be useful.

At very low pre-test likelihoods of either candidemia or intra-abdominal candidiasis, the practical value of negative test results is negligible. For example, a negative β-d-glucan in a setting that is low-risk for candidemia is predicted to reduce disease likelihood from ~1%–3% to <1%. At the same time, a β-d-glucan PPV of ~4%–11% is likely to be too low to justify treatment without a positive culture or other evidence of disease. At some high-end pre-test likelihood, NPVs become too low to be useful clinically. The NPV of β-d-glucan among high-risk GI surgery patients (pre-test likelihood ~30%) is only 78%, meaning clinicians must be willing to forego treatment despite a >20% chance that intra-abdominal candidiasis is present. Likewise, it is not clear that the 51% PPV of β-d-glucan in these settings has greater practical value than knowing the pre-test likelihood. At some point, the pre-test likelihood of invasive candidiasis may be sufficient to justify antifungal treatment regardless of non-culture test results. Indeed, universal prophylaxis is beneficial among groups such as bone marrow transplant recipients, surgical patients with recurrent GI leaks, and high-risk liver transplant recipients with bile leaks (≥30% incidence of Candida infection) [7,17].

**Figure 2:**
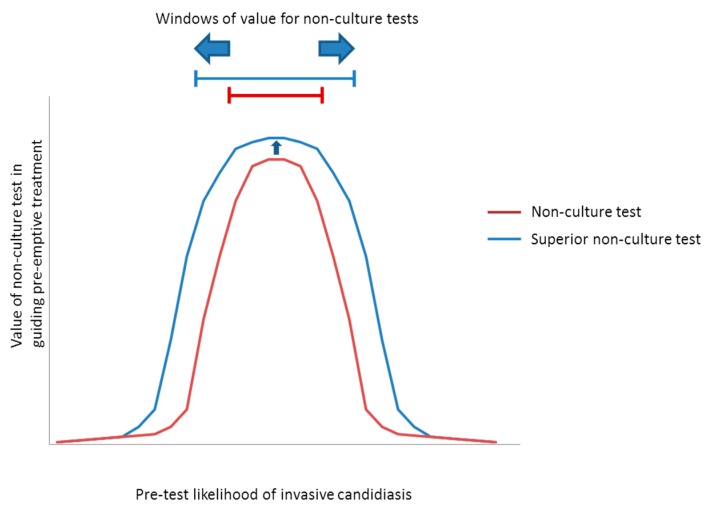
Schematic representation of the value of non-culture diagnostic tests in guiding antifungal treatment strategies. Data from Table 2 can be applied to the paradigm of Figure 1 to identify the range of pre-test likelihoods (windows) in which non-culture tests are predicted to be useful. If tests are performed in settings with very low incidence of invasive candidiasis, PPVs generally are too low to justify antifungal treatment and NPVs do not add significant marginal value over simply knowing the pre-test likelihood. As pre-test likelihood increases, improved PPVs hit a threshold at which pre-emptive treatment is beneficial. NPVs remain sufficiently high that antifungal treatment can be deferred without undue probability that invasive candidiasis is present. At some higher pre-test likelihood, however, NPVs hit a threshold at which the probability of invasive candidiasis is too high to forego antifungal treatment. The window in which non-culture tests promote pre-emptive treatment is defined by the pre-test likelihoods associated with threshold PPVs and NPVs (represented by bars in figure). A superior test (represented in blue) broadens the window compared to an inferior test (represented in red) by improving PPV and/or NPV, thereby dropping the low-end and/or raising the high-end pre-test likelihood (as shown by horizontal blue arrows). A superior test also has greater value in screening due to its higher PPV and/or NPV at a given pre-test likelihood (as shown by small vertical blue arrow).

If data from Table 2 are applied to the paradigm, windows of pre-test likelihoods can be assigned in which non-culture tests are most likely to be valuable in guiding pre-emptive or prophylactic treatment (Figure 2). Conclusions that may be drawn from this exercise are summarized in Table 3. The improved performance of a hypothetical PCR assay over β-d-glucan should expand the populations for antifungal treatment. PCR is expected to have greatest impact among those at-risk for candidemia, as a pre-emptive or prophyactic strategy becomes viable for lower-risk ICU patients (e.g., ICU resident for ≥4 days) and patients in septic shock. PCR is also predicted to extend treatment to patients at highest-risk for intra-abdominal candidiasis. An ideal non-culture test would diagnose both candidemia and intra-abdominal candidiasis with sensitivity/specificity of ~90%/99% (in other words, similar to the performance of T2Candida in preliminary studies of candidemia). A test with this performance for intra-abdominal candidiasis, rather than 80%/70% sensitivity/specificity suggested by the PCR literature, would expand the window to include lower-risk populations such as surgical ICU residents and peritoneal dialysis patients with peritonitis.

At pre-test likelihoods below the low-end of the window, the likely best management strategy is to defer testing and not initiate antifungal treatment. At pre-test likelihoods beyond the high-end of the window, the likely best strategy is to administer universal antifungal prophylaxis. Precise threshold PPV and NPV will need to be clarified in prospective clinical trials. Studies also are needed on the impact of antifungal treatment on non-culture test performance, which will have implications for prophylaxis strategies.

### 5.2. Treating Individual Patients

The use of non-culture tests in caring for individuals is more nuanced than when screening populations. Bayesian reasoning demands that any result be interpreted in some prior context [41,42]. In a given patient, “context” encompasses factors such as presenting complaints, underlying conditions, medications, physical findings, imaging studies and laboratory data, and the likelihood or exclusion of alternative etiologies. Each factor is a “result” that carries its own likelihood ratio and adjusts the probability of invasive candidiasis [41]. Even if each result has a low likelihood ratio, cumulatively they achieve a more powerful effect equal to the product of individual ratios. Therefore, moderate-risk ICU patients identified by clinical predictive scores (Mycoses Study Group criteria, as an example) may have very different pre-test likelihoods of candidemia at the time of testing, even though scores by themselves assign comparable risk (~10% incidence). The post-test likelihood may be further modified by the magnitude of results; two highly positive results are more compelling than a single borderline result. It is infeasible for clinicians to calculate precise running tallies of likelihood ratios in each patient. Nevertheless, they can conceptualize pre-test and post-test probabilities qualitatively [41]. Examples of useful qualitative evaluations include “my patient is reasonably likely to have invasive candidiasis, and a positive result significantly increases that likelihood”, or “in this low-risk patient, a positive result does not help me, but a negative result essentially excludes the disease”.

Pre-test likelihood windows that are important when screening populations are less relevant in an individual patient. A positive non-culture result with predictive value below a threshold nevertheless may justify antifungal treatment of a sick patient if there is no alternative diagnosis. Likewise, there is often clinical value in excluding invasive candidiasis, even if the pre-test likelihood is not within the screening window.

## 6. Conclusions

The interpretive framework and management strategies proposed here must be validated in clinical trials that assess endpoints such as rates of disease, patient outcomes, antifungal usage and resistance, and economic costs. Much-needed T2Candida data will be available soon from a multi-center study of patients with candidemia. In designing future trials of T2Candida, Fungitell or other assays, particular attention should be paid to blood culture-negative, intra-abdominal or deep-seated candidiasis. In addition, greater insight is needed into the optimal timing and frequency of testing in various populations, impact of prior or ongoing antifungal therapy, and assay kinetics in response to antifungal treatment. In the longer term, there is need for multi-plex assays, capable of detecting Candida, other pathogens, and markers for drug resistance such as *FKS* gene mutations [4,43].

In the era of molecular medicine, infectious diseases practitioners increasingly will be called upon to think and practice as Bayesians. In this regard, the diagnosis of invasive candidiasis is a case study for the types of challenges that will be encountered more widely in the not-so-distant future.

## References

[1] Andes D.R., Safdar N., Baddley J.W., Playford G., Reboli A.C., Rex J.H., Sobel J.D., Pappas P.G., Kullberg B.J. (2012). Impact of treatment strategy on outcomes in patients with candidemia and other forms of invasive candidiasis: A patient-level quantitative review of randomized trials. Clin. Infect. Dis..

[2] Morrell M., Fraser V.J., Kollef M.H. (2005). Delaying the empiric treatment of Candida bloodstream infection until positive blood culture results are obtained: A potential risk factor for hospital mortality. Antimicrob Agents Chemother..

[3] Garey K.W., Rege M., Pai M.P., Mingo D.E., Suda K.J., Turpin R.S., Bearden D.T. (2006). Time to initiation of fluconazole therapy impacts mortality in patients with candidemia: A multi-institutional study. Clin. Infect. Dis..

[4] Clancy C.J., Nguyen M.H. (2013). Finding the “missing 50%” of invasive candidiasis: How nonculture diagnostics will improve understanding of disease spectrum and transform patient care. Clin. Infect. Dis..

[5] Clancy C.J., Nguyen M.H. (2014). Undiagnosed invasive candidiasis: Incorporating non-culture diagnostics into rational prophylactic and preemptive antifungal strategies. Expert Rev. Anti Infect. Ther..

[6] Nguyen M.H., Wissel M.C., Shields R.K., Salomoni M.A., Hao B., Press E.G., Shields R.M., Cheng S., Mitsani D., Vadnerkar A. (2012). Performance of Candida real-time polymerase chain reaction, β-d-glucan assay, and blood cultures in the diagnosis of invasive candidiasis. Clin. Infect. Dis..

[7] Tissot F., Lamoth F., Hauser P.M., Orasch C., Fluckiger U., Siegemund M., Zimmerli S., Calandra T., Bille J., Eggimann P. (2013). β-glucan antigenemia anticipates diagnosis of blood culture-negative intraabdominal candidiasis. Am. J. Respir. Crit. Care Med..

[8] Calandra T., Bille J., Schneider R., Mosimann F., Francioli P. (1989). Clinical significance of Candida isolated from peritoneum in surgical patients. Lancet.

[9] Leroy O., Gangneux J.P., Montravers P., Mira J.P., Gouin F., Sollet J.P., Carlet J., Reynes J., Rosenheim M., Regnier B. (2009). Epidemiology, management, and risk factors for death of invasive Candida infections in critical care: A multicenter, prospective, observational study in france (2005–2006). Crit. Care Med..

[10] Delaloye J., Calandra T. (2014). Invasive candidiasis as a cause of sepsis in the critically ill patient. Virulence.

[11] Michalopoulos A.S., Geroulanos S., Mentzelopoulos S.D. (2003). Determinants of candidemia and candidemia-related death in cardiothoracic icu patients. Chest.

[12] Kett D.H., Azoulay E., Echeverria P.M., Vincent J.L. (2011). Candida bloodstream infections in intensive care units: Analysis of the extended prevalence of infection in intensive care unit study. Crit. Care Med..

[13] Ostrosky-Zeichner L., Sable C., Sobel J., Alexander B.D., Donowitz G., Kan V., Kauffman C.A., Kett D., Larsen R.A., Morrison V. (2007). Multicenter retrospective development and validation of a clinical prediction rule for nosocomial invasive candidiasis in the intensive care setting. Eur. J. Clin. Microbiol. Infect. Dis..

[14] Hadley S., Lee W.W., Ruthazer R., Nasraway S.A. (2002). Candidemia as a cause of septic shock and multiple organ failure in nonimmunocompromised patients. Crit. Care Med..

[15] Ostrosky-Zeichner L., Shoham S., Vazquez J., Reboli A., Betts R., Barron M.A., Schuster M., Judson M.A., Revankar S.G., Caeiro J.P. (2014). Msg-01: A randomized, double-blind, placebo-controlled trial of caspofungin prophylaxis followed by preemptive therapy for invasive candidiasis in high-risk adults in the critical care setting. Clin. Infect. Dis..

[16] Paphitou N.I., Ostrosky-Zeichner L., Rex J.H. (2005). Rules for identifying patients at increased risk for Candidal infections in the surgical intensive care unit: Approach to developing practical criteria for systematic use in antifungal prophylaxis trials. Med. Mycol..

[17] Eschenauer G.A., Kwak E.J., Humar A., Potoski B.A., Clarke L.G., Shields R.K., Abdel-Massih R., Silveira F.P., Vergidis P., Clancy C.J. (2014). Targeted versus universal antifungal prophylaxis among liver transplant recipients. Am. J. Transplant..

[18] Matuszkiewicz-Rowinska J. (2009). Update on fungal peritonitis and its treatment. Perit Dial. Int..

[19] Hall A.M., Poole L.A., Renton B., Wozniak A., Fisher M., Neal T., Halloran C.M., Cox T., Hampshire P.A. (2013). Prediction of invasive Candidal infection in critically ill patients with severe acute pancreatitis. Crit. Care.

[20] Mylonakis E., Clancy C.J., Ostrosky-Zeichner L., Garey K.W., Alangaden G.J., Vazquez J.A., Groeger S.J., Judson M.A., Vinagre Y.M., Heard S.O. (2015). T2 magnetic resonance assay for the rapid diagnosis of candidemia in whole blood: A clinical trial. Clin. Infect. Dis..

[21] Karageorgopoulos D.E., Vouloumanou E.K., Ntziora F., Michalopoulos A., Rafailidis P.I., Falagas M.E. (2011). β-d-glucan assay for the diagnosis of invasive fungal infections: A meta-analysis. Clin. Infect. Dis..

[22] He S., Hang J.P., Zhang L., Wang F., Zhang D.C., Gong F.H. (2015). A systematic review and meta-analysis of diagnostic accuracy of serum 1,3-β-d-glucan for invasive fungal infection: Focus on cutoff levels. J. Microbiol. Immunol. Infect..

[23] Neely L.A., Audeh M., Phung N.A., Min M., Suchocki A., Plourde D., Blanco M., Demas V., Skewis L.R., Anagnostou T. (2013). T2 magnetic resonance enables nanoparticle-mediated rapid detection of candidemia in whole blood. Sci. Transl. Med..

[24] Avni T., Leibovici L., Paul M. (2011). PCR diagnosis of invasive candidiasis: Systematic review and meta-analysis. J. Clin. Microbiol..

[25] Akamatsu N., Sugawara Y., Kaneko J., Tamura S., Makuuchi M. (2007). Preemptive treatment of fungal infection based on plasma (1→3)β-d-glucan levels after liver transplantation. Infection.

[26] Alexander B.D., Smith P.B., Davis R.D., Perfect J.R., Reller L.B. (2010). The (1,3)β-d-glucan test as an aid to early diagnosis of invasive fungal infections following lung transplantation. J. Clin. Microbiol..

[27] Singh N., Winston D.J., Limaye A.P., Pelletier S., Safdar N., Morris M.I., Meneses K., Busuttil R.W., Wagener M.M., Wheat L.J. (2015). Performance characteristics of galactomannan and β-d-glucan in high-risk liver transplant recipients. Transplantation.

[28] Pfeiffer C.D., Samsa G.P., Schell W.A., Reller L.B., Perfect J.R., Alexander B.D. (2011). Quantitation of *Candida* CFU in initial positive blood cultures. J. Clin. Microbiol..

[29] Telenti A., Steckelberg J.M., Stockman L., Edson R.S., Roberts G.D. (1991). Quantitative blood cultures in candidemia. Mayo Clin. Proc..

[30] Kiehn T.E., Wong B., Edwards F.F., Armstrong D. (1983). Comparative recovery of bacteria and yeasts from lysis-centrifugation and a conventional blood culture system. J. Clin. Microbiol..

[31] Brannon P., Kiehn T.E. (1985). Large-scale clinical comparison of the lysis-centrifugation and radiometric systems for blood culture. J. Clin. Microbiol..

[32] Muldoon E.G., Denning D.W. (2014). Editorial commentary: Prophylactic echinocandin: Is there a subgroup of intensive care unit patients who benefit?. Clin. Infect. Dis..

[33] Knitsch W., Vincent J.L., Utzolino S., Francois B., Dinya T., Dimopoulos G., Ozgunes I., Valia J.C., Eggimann P., Leon C. (2015). A randomized, placebo-controlled trial of preemptive antifungal therapy for the prevention of invasive candidiasis following gastrointestinal surgery for intra-abdominal infections. Clin. Infect. Dis..

[34] Playford E.G., Webster A.C., Sorrell T.C., Craig J.C. (2006). Antifungal agents for preventing fungal infections in non-neutropenic critically ill and surgical patients: Systematic review and meta-analysis of randomized clinical trials. J. Antimicrob Chemother..

[35] Shorr A.F., Chung K., Jackson W.L., Waterman P.E., Kollef M.H. (2005). Fluconazole prophylaxis in critically ill surgical patients: A meta-analysis. Crit. Care Med..

[36] Eggimann P., Francioli P., Bille J., Schneider R., Wu M.M., Chapuis G., Chiolero R., Pannatier A., Schilling J., Geroulanos S. (1999). Fluconazole prophylaxis prevents intra-abdominal candidiasis in high-risk surgical patients. Crit. Care Med..

[37] Garbino J., Lew D.P., Romand J.A., Hugonnet S., Auckenthaler R., Pittet D. (2002). Prevention of severe Candida infections in nonneutropenic, high-risk, critically ill patients: A randomized, double-blind, placebo-controlled trial in patients treated by selective digestive decontamination. Intensive Care Med..

[38] Pelz R.K., Hendrix C.W., Swoboda S.M., Diener-West M., Merz W.G., Hammond J., Lipsett P.A. (2001). Double-blind placebo-controlled trial of fluconazole to prevent Candidal infections in critically ill surgical patients. Ann. Surg..

[39] Goodman J.L., Winston D.J., Greenfield R.A., Chandrasekar P.H., Fox B., Kaizer H., Shadduck R.K., Shea T.C., Stiff P., Friedman D.J. (1992). A controlled trial of fluconazole to prevent fungal infections in patients undergoing bone marrow transplantation. N. Engl. J. Med..

[40] Slavin M.A., Osborne B., Adams R., Levenstein M.J., Schoch H.G., Feldman A.R., Meyers J.D., Bowden R.A. (1995). Efficacy and safety of fluconazole prophylaxis for fungal infections after marrow transplantation—A prospective, randomized, double-blind study. J. Infect. Dis..

[41] Gill C.J., Sabin L., Schmid C.H. (2005). Why clinicians are natural Bayesians. BMJ.

[42] Pauker S.G., Kopelman R.I. (1992). Interpreting hoofbeats: Can bayes help clear the haze?. N. Engl. J. Med..

[43] Shields R.K., Nguyen M.H., Press E.G., Kwa A.L., Cheng S., Du C., Clancy C.J. (2012). The presence of an *FKS* mutation rather than MIC is an independent risk factor for failure of echinocandin therapy among patients with invasive candidiasis due to *Candida glabrata*. Antimicrob. Agents Chemother..

